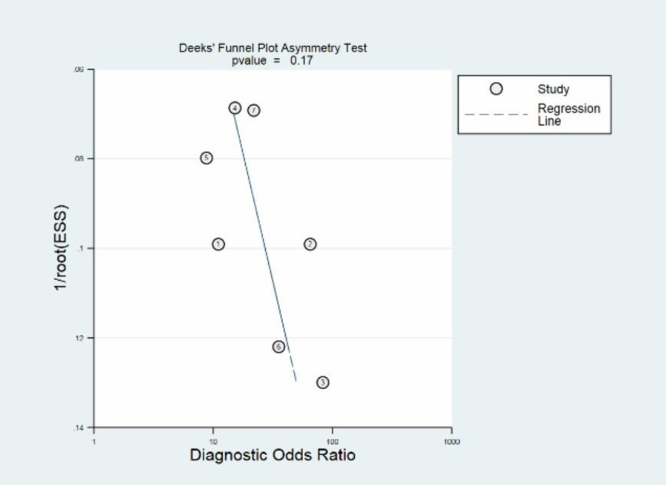# Comparative analysis of frailty identification tools in community services across the Asia-Pacific: A systematic review and meta-analysis

**DOI:** 10.1016/j.jnha.2025.100496

**Published:** 2025-01-30

**Authors:** Yi-Chen Wu, Chia-Te Chen, Shu-Fen Shen, Liang-Kung Chen, Li-Ning Peng, Heng-Hsin Tung

**Affiliations:** a College of Nursing and Health Sciences, Da-Yeh University, No.168, University Rd., Dacun, Changhua 515006, Taiwan; b Graduate Institute of Clinical Nursing, College of Medicine, National Chung Hsing University, No. 145 Xingda Rd., South Dist., Taichung City 402202, Taiwan; c Department of Nursing, College of Medicine, National Cheng Kung University, No.1-3, Daxue Rd., East Dist., Tainan City 70101, Taiwan; d Department of Nursing, Mackay Medical College, No.46, Sec. 3, Zhongzheng Rd., Sanzhi Dist., New Taipei City 252, Taiwan; e Center for Geriatrics and Gerontology, Taipei Veterans General Hospital, Taipei, Taiwan. No.201, Sec. 2, Shipai Rd., Beitou District, Taipei City, 11217, Taiwan; f Center for Healthy Longevity and Aging Sciences, National Yang Ming Chiao Tung University, No. 155, Sec.2, Linong Street, Beitou Dist., Taipei, 112, Taiwan; g Taipei Municipal Gan-Dau Hospital, No. 12, Ln. 225, Zhixing Rd., Beitou Dist., Taipei 112020, Taiwan; h College of Nursing, National Yang Ming Chiao Tung University, No. 155, Sec.2, Linong Street, Beitou Dist., Taipei, 112, Taiwan; i Tungs’ Taichung MetroHarbor Hospital, Taiwan, No.699, Section 8, Taiwan Boulevard, Wuqi District, Taichung City 435403, Taiwan

**Keywords:** Frailty identification, Instrument, Multiple tests, Multiple thresholds, Diagnostic test accuracy

## Abstract

**Objectives:**

This study aimed to compare various frailty screening and assessment tools with the Frailty Phenotype (FP), Frailty Index (FI), and Comprehensive Geriatric Assessment (CGA), which are considered the current gold standards, among the Asia-Pacific population in community settings.

**Design:**

Systematic review and meta-analysis.

**Setting and participants:**

This review included studies evaluating frailty identification tools based on the criteria of population, index and reference tests, and diagnosis of interest.

**Methods:**

A diagnostic test accuracy review was conducted to assess frailty instruments recommended by the Asia-Pacific Clinical Practice Guidelines. Comprehensive electronic database searches and manual searches were conducted up to August 20, 2024. Study quality, including risks of bias and applicability, was assessed using the QUADAS-2 tool. Hierarchical analysis and Youden’s index were employed to identify the optimal tool and cutoff points, and pooled frailty prevalence was calculated.

**Results:**

Fourteen studies were included: 10 for the FRAIL scale, 3 for TUG, and 2 for the SOF index (screening tools), and 2 each for the CFS and KCL, and 1 for the REFS (assessment tools). All studies demonstrated a high risk of bias. The pooled sensitivity and specificity for screening tools were 0.63 and 0.89, respectively, whereas for assessment tools, they were 0.79 and 0.85. The pooled prevalence of frailty and pre-frailty was 19.7% and 31.7%, respectively. The pooled diagnostic odds ratios were highest for the FRAIL scale (15.72) and CFS (35.03) among the screening and assessment tools. The subgroup analysis revealed that the setting had no significant impact on screening tool performance (p = 0.58), but a borderline significant effect was observed for assessment tools (p = 0.06), although this result is limited by the small number of studies, with only one conducted in a community setting. The FRAIL scale, with a cutoff of 2, had a Youden’s index of 0.60, signifying optimal screening performance.

**Conclusion:**

Among the frailty instruments recommended by the Asia-Pacific Clinical Practice Guidelines, this meta-analysis identifies the FRAIL scale as the most robust tool for distinguishing frailty, with a cutoff of 2 significantly enhancing diagnostic accuracy. Furthermore, the estimated prevalence of frailty in the Asia-Pacific region is 19.7% across various community settings, underscoring the need for further research and the development of validated assessment tools tailored to this population.

## Highlights

• Frailty screening and assessment tools showed good, though not perfect, accuracy.
• The FRAIL scale, with a cutoff of 2, indicates optimal screening performance.
• The pooled prevalence of frailty and pre-frailty was 19.7% and 31.7%, respectively.

## 1 Introduction

Frailty is recognized as a significant public health issue and a key challenge in modern geriatric care, particularly among older adults [1]. The International Association of Gerontology and Geriatrics Frailty Consensus defines frailty as a condition characterized by reduced strength and physiological dysfunction, which increases susceptibility to dependency, vulnerability, and a higher risk of mortality [2]. Despite substantial efforts by the scientific community over the past two decades to establish a clear definition of frailty, no consensus has yet been reached on a gold standard method for either screening or assessment [3]. Two studies revealed that, at that time, 67 different published methods for identifying frailty were in use [4,5]. The prevalence of frailty among community-dwelling older adults in the Asia-Pacific region varies widely, ranging from approximately 7.0%–65%, depending on the assessment tools employed and the inclusion of socioeconomically disadvantaged and indigenous communities [6,7]. This figure may also be underestimated due to the significant number of nonresponses in epidemiological surveys [8]. Moreover, due to the absence of specific acute physiological manifestations, frailty—a key focus of healthy aging—is often overlooked in both clinical and community settings, leading to missed opportunities for early screening, diagnosis, and prevention [9,10].

For the rapid and precise identification of frailty, the Asia-Pacific Clinical Practice Guidelines (CPGs) strongly advocate the use of validated measurement tools, such as Fried’s Frailty Phenotype (FP) and Rockwood and Mitnitski’s Frailty Index (FI) for expedited screening in clinical settings, alongside the Comprehensive Geriatric Assessment (CGA) for assessment in accurate diagnosis [[11], [12], [13], [14]]. A variety of frailty models and identifying tools exist, each differing considerably in terms of the time, training, and equipment required, as well as their applicability to specific clinical outcomes [15,16]. Furthermore, the selection of diagnostic tools should be informed by the resources available and their relevance to the specific clinical context [14]. Additionally, the cut-off points for physical assessments, used to diagnose frailty, should be tailored to populations within the Asia-Pacific region [15], as grip strength is generally lower compared to their European counterparts [17,18]. Consequently, it is advised to employ the lowest 20th percentile of grip strength as the benchmark for low muscle strength, with thresholds established at less than 26 kg for men and less than 18 kg for women [19].

In this context, utilizing a diagnostic test accuracy (DTA) analysis in conjunction with a meta-analysis presents a novel and compelling approach for providing a comprehensive evaluation of test accuracy in identifying frailty within the Asia-Pacific population. This method enables a more robust comparison of various tests, thereby offering stronger evidence in this field [20]. Moreover, a specific pooled DTA analysis would build upon existing evidence and provide valuable insights for stakeholders and policymakers in healthcare agencies, aiding in the decision-making process regarding the efficacy of frailty identification in primary clinical practice and community services, and supporting the implementation of preventive strategies in clinical settings [14,21].

While the tools recommended in the 2017 Asia Pacific CPGs are ubiquitous and widely used, there is limited evidence systematically evaluating their diagnostic performance within the Asia-Pacific context. This study aims to conduct a systematic review and diagnostic test accuracy (DTA) analysis to compare alternative frailty screening and assessment tools with the Frailty Phenotype (FP), Frailty Index (FI), and Comprehensive Geriatric Assessment (CGA), which are considered the current gold standards. The analysis focuses on the Asia-Pacific population within primary clinical practice and community service settings, evaluating the diagnostic accuracy of these tools in populations where socio-demographic, genetic, and healthcare system factors may influence their reliability. Additionally, the review considers the varying prevalence of conditions across the region, which may depend on the specific assessment tools utilized. This approach addresses critical evidence gaps related to region-specific diagnostic challenges and aims to provide insights that better inform clinical decision-making. Furthermore, it seeks to refine existing guidelines by evaluating whether the current recommendations are as effective in the Asia-Pacific population as they are in other regions where most studies originate.

## 2 Methodology

In developing the study design, we consulted the Preferred Reporting Items for Systematic Reviews and Meta-Analyses 2020 statement (PRISMA 2020) principles [22] which is an updated set of guidelines from PRISMA 2009, and the Cochrane Handbook for Systematic Reviews of Diagnostic Test Accuracy [23]. The study is registered at PROSPERO (ID: CRD42024578685).

### 2.1 Selection criteria

Studies were selected based on population, index test, reference test, and diagnosis of interest (PIRD) criteria for diagnostic test accuracy reviews [24]. The population criteria for the included studies were community-dwelling older adults with a minimum mean age of 65 years, or where at least half of the study participants were aged ≥65 years. Additionally, the studies were conducted in the Asia-Pacific region, which is geographically defined as encompassing countries in East Asia, Southeast Asia, and Oceania that are adjacent to or located within the western Pacific Ocean. This includes China, Japan, Korea, Taiwan, Singapore, Thailand, Australia, New Zealand, and other nations. Studies focused on specific diagnoses or conducted in acute settings (e.g., chronic kidney disease, heart failure, diabetes mellitus, cancer, surgical patients, emergency department, or hospitalized patients) were excluded. Instruments recommended by the Asia-Pacific CPGs for the management of frailty, used for identifying frailty in either screening (e.g., the FRAIL scale, the Study of Osteoporotic Fractures (SOF) index, the Short Physical Performance Battery (SPPB), and the Timed-Up-and-Go (TUG) test) or assessment (e.g., the Clinical Frailty Scale (CFS), the Kihon Checklist (KCL), and the Reported Edmonton Frailty Scale (REFS)), were defined as the index tests for this review. The instruments selected for inclusion in our analysis were determined based on the degrees of recommendation outlined in the Asia-Pacific Clinical Practice Guidelines. While the CPGs references more than 20 frailty tools, only those with strong recommendations and robust evidence supporting their validity, reliability, and practicality within the Asia-Pacific population were included. This selection process was designed to ensure that the tools analyzed were the most relevant and applicable to the objectives of this study. Studies that assessed the DTA of one or more index tests against one or more of the three frailty reference standards—frailty phenotype (FP) [11], Frailty Index (FI) [12], or specialist comprehensive geriatric assessment (CGA) [13,25]—were considered for inclusion. Detailed information about the included index tests and reference standards is presented in Table 1. The diagnosis of interest was frailty.

**Table 1:** Overview of the including index test.

Index		Description
Screening		
	FRAIL scale	The FRAIL scale is a brief five-item assessment used to screen for frailty. The items include Fatigue, Resistance (difficulty climbing stairs), Ambulation (slow walking speed), Illness (number of chronic conditions), and Loss of weight (≥ 5% in the 6 months). Each "Yes" response is scored as 1 point, while a "No" response is scored as 0. Based on the total score, individuals are classified as robust (score = 0), prefrail (score = 1–2), or frail (score = 3–5) [2,54]. The FRAIL scale has been demonstrated to be an effective screening tool for clinicians to identify individuals at risk of health decline and increased mortality [51,52].
	Study of Osteoporotic Fractures (SOF) index	The Study of Osteoporotic Fractures (SOF) index was initially developed as part of a large cohort study aimed at identifying risk factors for fractures and other health outcomes in older women [57]. This simplified index consists of three easily measurable components: unintentional weight loss, difficulty rising from a chair five times without using the arms, and low energy levels. It is designed to identify phenotypic frailty in community-dwelling older adults, particularly in busy primary care settings. Individuals with none of these components are classified as robust, those with one component are considered prefrail, and those with two or three components are categorized as frail. The SOF index has been shown to be an independent predictor of adverse health outcomes in community-dwelling older adults and generally compares favorably in risk prediction to other frailty indices [58].
	Short Physical Performance Battery (SPPB)	The Short Physical Performance Battery (SPPB) is a tool used to assess lower extremity function in older adults. It consists of three components: balance (side-by-side, semi-tandem, and tandem stands), gait speed (time to walk 8 feet), and strength (time to rise from a chair five times). Each component is scored from 0 to 4, with a total possible score of 12. Higher scores indicate better physical performance [59]. The SPPB is associated with adverse outcomes such as falls, hospitalization, and mortality. Moreover, it takes less than 10 min to administer and has been validated as a reliable measure of physical function in older adults across diverse populations [60].
	Timed-Up-and-Go (TUG)	The Timed Up and Go (TUG) test is a simple tool used to measure mobility, balance, and functional ability, particularly in older adults. It evaluates how quickly a person can stand up from a chair, walk a short distance, turn around, and return to the chair following instructions. The time it takes to complete this task is recorded. The cutoffs are 10 seconds for good mobility, 10–20 seconds for impaired mobility, and over 20 seconds for poor mobility [61]. The TUG is a simple, quick, easy, and validated tool that captures components of frailty, which become more common with age. It is used to assess fall risk, functional status, and to identify physical frailty and low physical performance [[62], [63], [64]].
Assessment		
	Clinical Frailty Scale (CFS)	The Clinical Frailty Scale (CFS), developed by Dr. Kenneth Rockwood and colleagues in 2005 as part of the Canadian Study of Health and Aging [65], is a widely used tool for assessing frailty in older adults. The CFS uses clinical judgment to evaluate physical function, comorbidities, cognitive status, and the ability to perform activities of daily living (ADLs). It categorizes individuals on a 9-point scale, from 1 (very fit) to 9 (terminally ill). A person with a score ≥ of 5 is considered frail [66]. Commonly used in clinical and primary care settings, the CFS helps guide medical interventions by predicting outcomes like mortality, hospitalization, and postoperative complications. It has been validated as a strong predictor of health outcomes and is a reliable, practical tool when used consistently [67].
	Kihon Checklist (KCL)	The Kihon Checklist (KCL), developed by the Japanese Ministry of Health, Labour and Welfare, assesses frailty and identifies older adults at risk of functional decline [44]. Primarily used in community settings, it helps detect early signs of frailty and guides interventions to maintain independence. The KCL consists of 25 yes/no questions across seven domains: physical function, nutritional status, oral function, social activities, cognitive function, mood, and daily activities. Each "yes" response scores 1, with higher total scores indicating greater frailty risk [44]. The KCL is a simple, quick, and proven tool for identifying frailty and functional decline in community settings, enabling healthcare systems to recognize vulnerable older adults and provide timely interventions [68,69].
	Reported Edmonton Frailty Scale (REFS)	The Reported Edmonton Frailty Scale (REFS) is a modified version of the original EFS, designed to quickly assess frailty in older adults. It evaluates multiple health domains, including cognition, general health, functional independence, social support, medication use, mood, nutrition, continence, and functional performance, across 13 items. The maximum possible score is 18, with frailty classified as "severe frailty" (score 12–18), "moderate frailty" (score 10–11), "mild frailty" (score 8–9), "apparently vulnerable" (score 6–7), and "not frail" (score 0–5) [70]. The REFS is a simple, comprehensive tool that predicts adverse outcomes such as hospitalization, falls, disability, and mortality, making it valuable for early detection and guiding healthcare decisions in older adults [37,71].
Reference standards		
	Frailty Phenotype (FP)	The Frailty Phenotype (FP), developed by Linda Fried and colleagues, defines frailty as a clinical syndrome characterized by the presence of three or more of five specific criteria: unintentional weight loss, self-reported exhaustion, weakness (measured by grip strength), slow walking speed, and low physical activity. It is used to identify individuals at higher risk of adverse outcomes, such as falls, hospitalization, disability, and mortality, particularly among older adults (Fried et al., 2001). This tool is validated and commonly used in research and clinical settings to screen for physical frailty [14].
	Frailty Index (FI)	The Frailty Index (FI), developed by Rockwood and Mitnitski, conceptualizes frailty as the accumulation of health deficits, which may include symptoms, signs, diseases, and disabilities. It is calculated by dividing the number of observed deficits by the total number of possible deficits, resulting in a score that reflects an individual’s level of frailty, with higher scores indicating greater frailty [12]. The FI is a comprehensive, quantitative, and validated tool widely used in research and clinical settings to evaluate overall frailty risk and predict adverse outcomes, such as hospitalization, disability, and mortality [14].
	Comprehensive Geriatric Assessment (CGA)	The Comprehensive Geriatric Assessment (CGA) is a multidimensional, interdisciplinary diagnostic process designed to evaluate the medical, psychological, functional, and social needs of older adults. Conducted by a team of healthcare specialists, it aims to develop a coordinated care plan to improve health outcomes, optimize functional status, and enhance quality of life [25]. The validated CGA is often used in clinical settings to identify frailty, guide treatment decisions, and coordinate long-term care, with a focus on individualized, patient-centered care [14].

Given the nature of our research question, we included observational studies (e.g., cohort) and cross-sectional studies, as well as clinical trials (whether randomized or not). Access to the full-text article was required, and abstracts and publications in languages other than English were not eligible for inclusion. Animal studies, case reports or series, commentaries, opinion pieces, conference abstracts, editorials, protocol submissions, and review articles were excluded. Additionally, the reference lists of relevant review articles were searched for additional studies. Authors of excluded papers, including abstracts, were also contacted to inquire whether their data had been submitted for publication and, if so, whether it could be obtained in advance for potential inclusion in this review.

### 2.2 Study databases and searching strategy

PubMed, Medline, the Cochrane Library, CINAHL, Airiti Library and the google scholar were systematically searched from inception to August 20, 2024, to retrieve eligible studies that assessed frailty diagnosis using instruments recommended by the Asia-Pacific CPGs. The search strategy included the use of a combination of MeSH terms and keywords pertaining to frailty (“frail” OR “frailty”), diagnostic accuracy (“sensitivity” OR “specificity” OR “AUC” OR “ROC” OR “predictive value” OR “diagnostic accuracy” OR “diagnostic performance” OR “diagnostic utility”), AND the name of the reviewed index scale (“Clinical Frailty Scale” OR “CFS” OR “FRAIL scale” OR “Kihon checklist” OR “KCL” OR “Study of Osteoporotic Fractures index” OR “SOF index” OR “Short Physical Performance Battery” OR “SPPB” OR “Reported Edmonton Frailty Scale” OR “REFS” OR “Timed-Up-and-Go” OR “TUG”) And the name of the reviewed reference scale (“Fried's Frailty Phenotype” OR “FP” OR “Frailty Index” OR “FI” OR "Comprehensive Geriatric Assessment" OR "CGA"). Additional eligible studies were identified by manually searching the reference lists of all the included studies. All systematic literature searches were conducted independently by two researchers (YW and CC).

### 2.3 Study selection

All studies of interest were exported from the respective databases and imported into EndNote 20.1 bibliographic software (Thomson Reuters, San Francisco, CA, USA). Two researchers independently screened the titles and abstracts of each search result according to the eligibility criteria to select potential studies for inclusion. Duplicates were initially excluded during the import process into EndNote, and any remaining duplicates were manually removed by the researchers. Studies meeting the required criteria were then reviewed in full independently by the two researchers to decide on the final studies for inclusion. Lastly, they independently screened the citations in the included papers to identify other studies meeting the search criteria. Any disagreements were resolved by consensus.

### 2.4 Data extraction

Data were extracted from the accepted studies by two independent researchers (YW and CC) using standardized templates, following the guidance of Campbell et al. [26]. The data collected included:

• **Administrative Details**: Information such as author, year of publication, and country of origin.
• **Study Characteristics**: This encompassed the study design, study setting, sample size, and participant characteristics and demographics.
• **Details of the Frailty Assessment Instruments**: This included names of the frailty measurement tools and its cutoffs, tool adaptation and method of test administration.
• **Comparison Standards**: Identification of the "gold standard" against which the instruments were compared, such as FP (Frailty Phenotype), FI (Frailty Index), or CGA (Comprehensive Geriatric Assessment), and its cutoff.
• **Prevalence of Frailty**: The measured prevalence of frailty in the study populations.

Disagreements related to data extraction were discussed and resolved by consensus. If sensitivity, specificity, positive predictive value, or negative predictive value were not directly reported, these metrics were calculated using the study findings or, when necessary, additional information obtained from the authors. Missing or incomplete data were addressed by reaching out to the study authors, and any missing values were documented in the data extraction form.

### 2.5 Risk of bias assessment

The methodological standards of the accepted publications were independently evaluated by two researchers (YW and CC) using the Quality Assessment of Diagnostic Accuracy Studies-Comparative (QUADAS-C) tool, which is an extension of the QUADAS-2 tool designed to assess the risk of bias and diagnostic accuracy in comparative studies [27]. This tool comprises four key domains: patient selection, index test, reference standard, and flow and timing. Each domain was assessed for the risk of bias, and the first three domains were also evaluated for concerns regarding their applicability to the research question. Any disagreements between the reviewers were resolved through discussion and, if necessary, by consulting the third author (SS). A quality assessment summary of eligible studies was carried out using RevMan 5.4 (RevMan, 2014).

### 2.6 Data synthesis

The data analysis utilized both the original values for true positives, false positives, true negatives, and false negatives, as well as those calculated from the reported sensitivity and specificity. For studies that used the index test with multiple reference standards, the data for true positives, false positives, true negatives, and false negatives were split to address potential unit-of-analysis errors. The data analysis was completed in four steps: calculating diagnostic accuracy measures, performing statistical modeling or meta-analysis, assessing heterogeneity, and conducting bias and sensitivity analyses, following the summarization of the extracted data into a 2 × 2 table [28]. All analyses were performed using Stata/MP (vers. 15.0; StataCorp LLC, College Station, TX, USA).

Initially, a bivariate statistical analysis using a random-effects model was conducted to estimate the pooled sensitivity and specificity across studies for two categories of tools: screening and assessment. Sensitivity and specificity pairs from each included study were analyzed, accounting for potential threshold effects and the correlation between the two measures [21]. Forest plots were created to visualize the results. Significant heterogeneity among the included studies was assessed using Cochran’s *Q* test (p < .10) and *I*^2^ values, with cut-off points of 25%, 50%, and 75% representing low, medium, and high heterogeneity, respectively [29].

Second, since heterogeneity is inherent in DTA studies, hierarchical models were used to jointly analyze sensitivity and specificity, as these are the most statistically rigorous and recommended methods for addressing threshold effects [30]. The HSROC (Hierarchical Summary Receiver Operating Characteristic) curve was generated after estimating the pooled sensitivity and specificity. It visualizes the results using summary points for sensitivity and specificity, along with their corresponding confidence and prediction regions. Moreover, the associated areas under the curves (AUCs) were estimated to measure overall diagnostic accuracy, reflecting the probability of correctly classifying a randomly selected participant as either a case or a control. According to the guidelines for interpreting AUC values, the diagnostic accuracy of a test is classified as low for AUC values between 0.5 and 0.7, moderate for values between 0.7 and 0.9, and high for values between 0.9 and 1.0 [31]. In addition, we applied a random-effects model to calculate the pooled frailty and pre-frailty prevalence, based on different cutoffs, with a 95% confidence interval (CI) when the *I²* index indicated high heterogeneity; otherwise, a fixed-effects model was used. In studies that evaluated frailty using different diagnostic indices, the prevalence most closely resembling the recommended cutoff from the Asia-Pacific CPGs was pooled in the meta-analysis.

Considering the variation in sample sizes and the number of studies in the context of DTA, meta-regression and subgroup analyses were conducted to explore the relationship between study-level covariates, such as the type of index test, and latent sensitivity and specificity [32]. Moreover, the diagnostic odds ratio (DOR) was estimated, combining sensitivity and specificity to assess the overall diagnostic performance of each individual scale. A higher DOR indicates that the odds of the test producing correct results are greater compared to the odds of producing incorrect results [33]. Additionally, Youden's index was calculated using the formula J = (sensitivity + specificity) − 1 to identify the diagnostic test and threshold that achieved the optimal balance between sensitivity and specificity for ranking the frailty measurement tools [34]. Maximizing Youden's index indicates the most effective test and diagnostic threshold for diagnosing a disease in a specific patient group [35]. Finally, Deeks' funnel plot asymmetry test, a regression test in which the DOR is plotted against the inverse of the square root of the effective sample size, was used to examine publication bias. A result of p < .10 indicates significant asymmetry, suggesting the presence of publication bias [36].

## 3 Results

### 3.1 Study selection

**Fig. 1:**
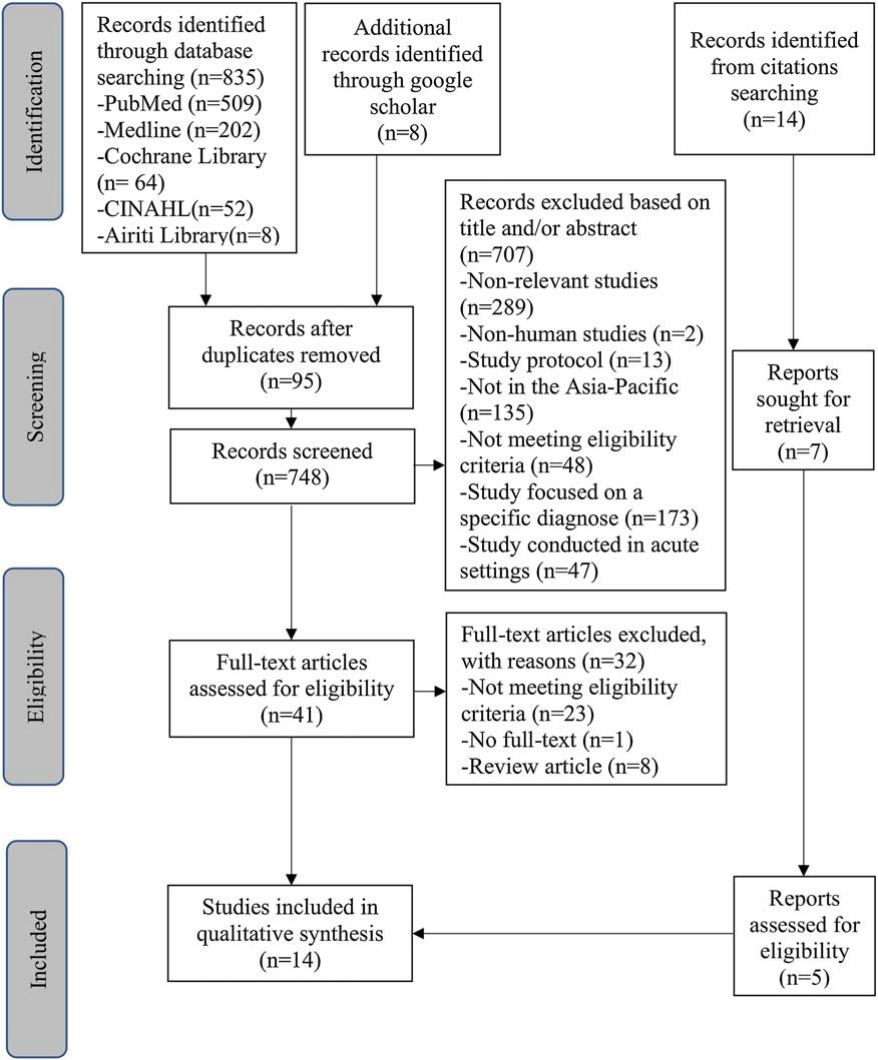
Flowchart of studies selection.

A total of 857 published articles were retrieved. After excluding duplicates, 748 articles were screened by their titles and abstracts for relevance to our inclusion criteria. Of these, 707 articles were rejected because they did not meet our inclusion criteria. The remaining 41 articles were selected for full-text retrieval to assess their eligibility for the final analysis. Among these, twenty-three studies used unsuitable reference standards, one study could not be retrieved in full-text, and eight were review articles. After reviewing the references of eligible studies, 14 additional studies were identified, resulting in a total of fourteen studies being included in the meta-analysis. The PRISMA chart illustrating the search results is shown in Fig. 1.

### 3.2 Study characteristics

Details of the included studies are presented in Table 2. Among the 14 studies, 10 examined the FRAIL scale, 3 examined the TUG test, and 1 examined the REFS. Additionally, 2 studies examined the SOF index, CFS, and KCL, while no eligible studies were found for the SPPB. The countries of origin were as follows: Australia [37,38], China [39,40], Indonesia [41,42], Japan [43,44], Singapore [45], South Korea [46,47], Taiwan [48], Thailand [49], and Vietnam [50]. Thirteen of these studies were conducted using a cross-sectional design in community-based or outpatient clinic settings. Overall, the included sample size was 7,186 and 729 in the frailty measurement tools of screening and assessment, respectively. The global mean ages of participants using the screening tools were 72.58 years for the FRAIL scale, 72.46 years for the TUG, and 70.65 years for the SOF index. In contrast, the mean ages for participants using the assessment tools were 72.28 years for the CFS, 77.7 years for the KCL, and 79 years for the REFS. Proportions of male participants in studies using frailty measurement tools of screening and assessment were 40.7%, and 51.8%, respectively. Moreover, 11 studies examined only one index test, while the other three studies [37,40,49] used two, three, and four index tests, respectively, among the 14 studies. Various cutoff points for the index tests were used in the screening tools, including FRAIL scale (≥1, ≥2, or ≥3), TUG (≥7.7 seconds, ≥8.2 seconds, or ≥10 seconds), and SOF index (≥1 or ≥2), as well as in the assessment tools, including CFS (≥4 or ≥5), KCL (3/4, 7/8, or ≥7), and REFS (≥8). Regarding the reference standard, 9 studies used FP, two studies used FI, one study used CGA, and two studies used both FP and FI. The cutoffs used to identify frailty were ≥3 in FP, ≥0.20 or ≥0.25 in FI, and ≥2 in CGA.

**Table 2:** Characteristics of included studies.

Index test	First author (year of publication)	Country of origin	Study design	Study setting	Participants’ inclusion Criteria	Sample size	Mean age ± SD (years)	Male (%)	Cutoff of index test	Tool Adaptation	Method of test administr-ation	Reference standard and cutoff	Preval-ence of Frailty
**SCREENING**													
FRAIL													
	Ambagtsheer (2020)	Australia	Cross-sectional	General Practice	Participants were aged 65 years older, living independently in the community, and attending general practices	228	79.0 (Median)	45.2%	≥3	No	Nurse-administered	FP ≥3	10.1%
	Anh (2022)	Vietnam	Cross-sectional	Geriatric clinic	Older patients, (≥60 years old) attending the Geriatric clinic, needed to be able to communicate and walk a distance of five meters	396	72.6 ± 7.6	37.9%	≥2, ≥3	Translated and culturally adapted FRAIL-VI	Self-administered or interviewer-administered	FP ≥3	21.7%
	Chen (2020)	Japan	Cross-sectional	Community-based	The study included older adults aged 65−75 years from the Itoshima Frail Study (IFS).	858	70.9 ± 3.2	48.3%	≥2, ≥3	Adapted FRAIL-J to Japanese context	Self-administered	FP ≥3	2.3%
	Dong (2018)	China	Cross-sectional	Urban communities	Community-dwelling older adults aged 60 years and older	1,235	69.46 ± 6.72	30.6%	≥2, ≥3	Translated and culturally adapted to Chinese	Face-to-face interviews by trained staff	FP ≥3	6.6%
	Dwipa (2021)	Indonesia	Cross-sectional	Geriatric clinic outpatients	Patients aged >60 years who could communicate in the Indonesian language and answer questions for themselves	101	79	56.4%	≥2, ≥3	Translated and culturally adapted to Indonesian	Face-to-face interviews by trained staff	FP ≥3	28.7%
	Jung (2016)	South Korea	Cross-sectional	Geriatric Center	Patients aged ≥65 years who underwent a comprehensive geriatric assessment, who needed to be able to verbally communicate or have a guardian closely involved in their care.	103	76.8 ± 6.1	53.4%	≥1, ≥3	Translated and culturally adapted to Korean	Administered during CGA with nurse's assistance	FI ≥0.20	17.5%
	Lim (2020)	Singapore	Cross-sectional	Senior Activity Centres	Older adults aged 60 years and older who were community-dwelling and attended community health centers	517	67.7 ± 6.9	26.9%	≥1, ≥3	Standard instruments with validated components	Administered by trained study team	FP≥1, ≥3, FI > 0.08, ≥0.25	1.35%
	Si (2021)	China	Cross-sectional	Community	Community-dwelling older adults aged 60 years and older	1,177	69.3 ± 6.6	30.3%	≥3	Translated and validated in Chinese context	Administered face-to-face by trained professionals	CGA ≥2	4.8%
	Sukkriang (2020)	Thailand	Cross-sectional	Community	Community-dwelling elderly aged 60 years and older	214	70.07 ± 7.15 (Males), 69.28 ± 7.13 (Females)	50%	≥3	Translated and culturally adapted to Thai context	Face-to-face by trained professionals	FP≥3	22.9%
	Thompson (2020)	Australia	Secondary analysis of a longitudinal population survey	Community	Community-dwelling older adults aged 65 years and older	846	74.3 ± 6.3	45.2%	≥1, ≥3	Modified from survey data	Retrospective construction from survey data	FP≥3	22.5%
TUG													
	Ambagtsheer (2020)	Australia	Cross-sectional	General Practice	Participants were aged 65 years older, living independently in the community, and attending general practices	228	79.0 (Median)	45.2%	≥10 seconds	No	Nurse-administered	FP ≥3	55.3 %
	Sukkriang (2020)	Thailand	Cross-sectional	Community	Community-dwelling elderly aged 60 years and older	214	70.07 ± 7.15 (Males), 69.28 ± 7.13 (Females)	50%	≥10 seconds	Translated and culturally adapted to Thai context	Face-to-face by trained professionals	FP≥3	23.8%
	Tang (2015)	Taiwan	Cross-sectional	Community	Adults aged 50 years and older who actively participated in local community programs	65	71.5 ± 8.1	35.4%	≥7.7 seconds (single) ≥8.2 seconds (manual)	Adapted for single-task and dual-task (manual and cognitive) assessment	Face-to-face	FP≥3	56.9%
SOF index													
	Seto (2015)	Indonesia	Cross-sectional	Geriatric Outpatient Clinic	Patients aged ≥60 years attending the Geriatric Outpatient Clinic	269	72 (Median)	39.4%	Not stated	No	Direct measurements	FI	25.3%
	Si (2021)	China	Cross-sectional	Community	Community-dwelling older adults aged 60 years and older	1,177	69.3 ± 6.6	30.3%	≥2	Translated and validated in Chinese context	Administered face-to-face by trained professionals	CGA ≥2	4.5%
**ASSESSMENT**													
CFS													
	Jung (2021)	South Korea	Cross-sectional	Geriatric outpatient clinic	Community-dwelling patients who were ambulatory with or without walking aids and were able to communicate with the examiners	123	77.49	45.5%	≥4	Korean version of CFS	Administered during CGA by nurses	FP≥3, FI≥0.25	29.3%
	Sukkriang (2020)	Thailand	Cross-sectional	Community	Community-dwelling elderly aged 60 years and older	214	70.07 ± 7.15 (Males), 69.28 ± 7.13 (Females)	50%	≥5	Translated and culturally adapted to Thai context	Face-to-face by trained professionals	FP≥3	7.9%
Kihon checklist													
	Ambagtsheer (2020)	Australia	Cross-sectional	General Practice	Participants were aged 65 years older, living independently in the community, and attending general practices	228	79.0 (Median)	45.2%	≥7	Culturally adapted	Self-administered	FP ≥3	36.8%
	Satake (2016)	Japan	Cross-sectional	Hospital outpatient clinic, National Center for Geriatrics and Gerontology	Elderly outpatients aged 65 years and older living independently, who attended regular check-ups for chronic conditions	164	76.4 ± 6.2	66.5%	3/4 (prefrailty), 7/8 (frailty)	No	Self-reports with nurse assistance	FP	11.6%
Reported Edmonton Frailty Scale													
	Ambagtsheer (2020)	Australia	Cross-sectional	General Practice	Participants were aged 65 years older, living independently in the community, and attending general practices	228	79.0 (Median)	45.2%	≥8	No	Self-administered	FP ≥3	35.5%

### 3.3 Study quality appraisal

The included studies, which used FRAIL scale, TUG, SOF index, CFS, KCL, and REFS, were assessed for quality using the QUADAS-2 tool. In the criterion of patient selection, four studies were rated as having unclear risk due to insufficient information about the selection method, while three studies were rated as having high risk, indicating that unsuitable study designs and participant eligibility criteria were used. The majority of studies were rated as having unclear or high risk for the index test (n = 11) and reference standard (n = 9) criteria, due to the unclear description of the sequence of interpretation between the index test and the reference standard. In the criterion of flow and timing, six studies were rated as having unclear risk because they did not clearly describe the interval between the index test and the reference standard, while two studies were rated as having high risk because they did not include all patients in the analysis. In terms of applicability concerns, although two studies were rated as having high risk in patient selection, the overall bias risk was low, indicating that the included studies adequately addressed the review questions. Appendix (Fig. 1A, B) illustrates the Cochrane risk of bias assessment for the included studies.

### 3.4 Meta-analysis

**Fig. 2:**
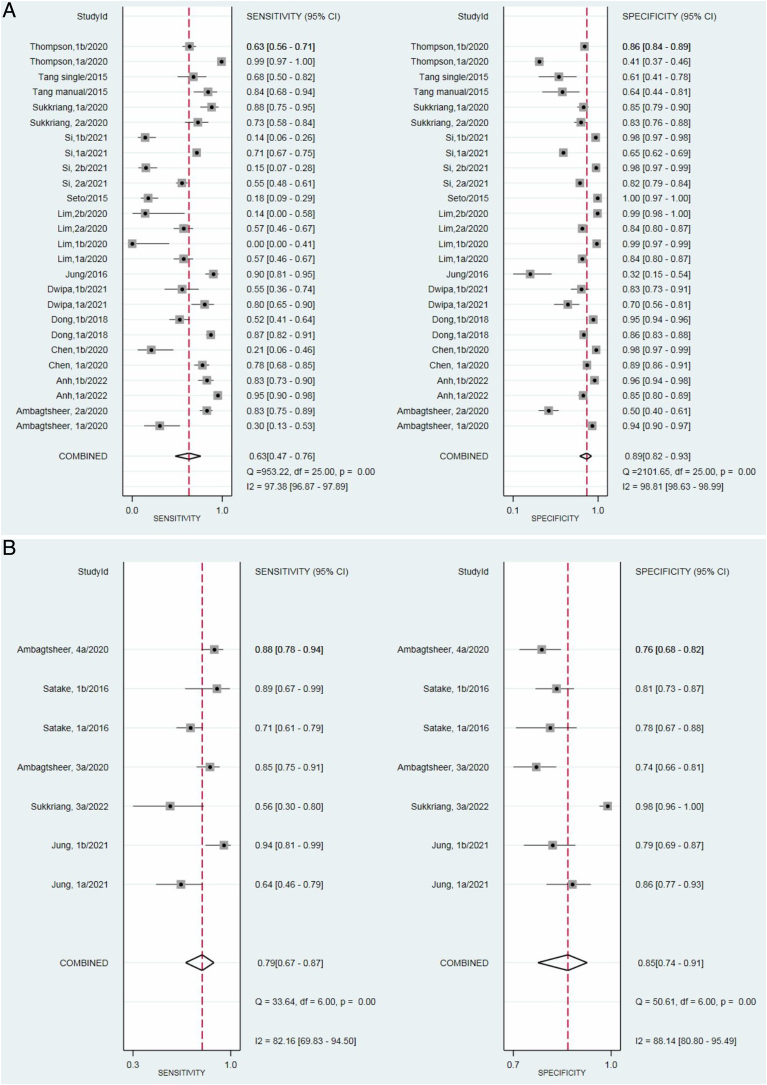
Paired forest plots of frailty measurement tools: in screening (A) and in assessment (B).

The preliminary step in assessing the heterogeneity of the studies, to guide further hierarchical analysis, involved using paired forest plots of sensitivity and specificity for the two categories of frailty measurement tools—screening and assessment—as depicted in Fig. 2. Among the 12 studies that evaluated the tools for screening, three studies conducted two index tests (i.e., FRAIL and TUG, SOF index and CGA), one study used two reference standards (i.e., FP and FI), and seven studies examined two cut-off points (i.e., ≥2 and ≥3); thus, 26 data points were used for the analysis. A random-effects model indicated that the pooled sensitivities and specificities of the tools for screening were 0.63 (95 % CI: 0.47−0.76) and 0.89 (95 % CI: 0.82−0.93), respectively. The heterogeneity test revealed substantial heterogeneity, as indicated by the *Q* statistic and *I²* index, for both pooled sensitivity (*Q* = 953.22, *p* < .001, *I*^2^ = 97.38) and pooled specificity (*Q* = 2101.65, *p* < .001, *I*^2^ = 98.81).

In terms of the tools for assessment, one study conducted two index tests (i.e., KCL and REFS), one study used two reference standards (i.e., FP and FI), and one study examined two cut-off points (i.e., 3/4 and 7/8); thus, 7 data points were used for analysis across the 4 studies. The pooled sensitivities and specificities of the tools for assessment were 0.79 (95 % CI: 0.67−0.87) and 0.85 (95 % CI: 0.74−0.91), respectively. The heterogeneity test revealed that the *Q* statistic and *I*^2^ index indicated substantial heterogeneity in the pooled sensitivity (*Q* = 33.64, *p* < .001, *I*^2^ = 82.16) and pooled specificity (*Q* = 50.61, *p* < .001, *I*^2^ = 88.14).

Given the considerable heterogeneity, likely due to the choice of cut-off points used in the studies, hierarchical analysis and HSROC curves were employed to estimate the pooled sensitivity and specificity. Appendix (Fig. 2A and B) illustrates both the pooled diagnostic performance (as indicated by the summary point) and the variability in diagnostic accuracy across studies. For the screening tools, the closer the HSROC curve is to the upper left corner, the better the diagnostic accuracy. Tight confidence and prediction regions suggest consistent performance across studies, while larger regions indicate greater heterogeneity. For the assessment tools, the HSROC curve shows moderate diagnostic accuracy, being near but not fully in the upper left corner. The tight confidence region suggests consistent findings, while the larger prediction region indicates variability in test performance across studies. In addition, the associated AUCs were 0.86 and 0.89 for screening and assessment tools, respectively.

The prevalence of frailty and pre-frailty ranged widely, from 1.3% to 56.9% and 12.5% to 60.4%, respectively, with pooled estimated values of 19.7% (*I*^2^ = 98.2%, 95% CI = 0.16–0.23) for frailty and 31.7% (*I*^2^ = 97.8%, 95% CI = 0.25–0.38) for pre-frailty, based on 8,941 and 7,606 participants across 12 and 14 studies, respectively, using the relevant cutoffs in each tool, which are available in Appendix (Fig. 3A and B).

### 3.5 Meta-regression and subgroup analysis

A meta-regression and subgroup analysis were conducted to assess whether the use of different instruments for identifying frailty affected sensitivity and specificity across studies, as well as to distinguish frailty between non-healthcare community settings and clinic settings. For screening tools, there was no statistically significant difference in pooled relative sensitivity for the FRAIL, SOF index, and TUG, with p-values of 0.79, 0.06, and 0.42, respectively, and no significant difference in pooled relative specificity for the FRAIL and SOF index, with p-values of 0.57 and 0.21, respectively. However, there was a statistically significant difference in pooled relative specificity for the TUG, with a p-value of 0.02. For assessment tools, there was no statistically significant difference in pooled relative sensitivity for the CFS, KCL, and REFS, with p-values of 0.14, 0.46, and 0.68, respectively, and no significant difference in pooled relative specificity for the CFS and REFS, with p-values of 0.88 and 0.37, respectively. However, there was a statistically significant difference in pooled relative specificity for the KCL, with a p-value of 0.04.

In addition, the pooled DORs were 15.72 (95% CI: 9.66, 25.58), 8.09 (95% CI: 3.80, 17.24), and 6.58 (95% CI: 3.56, 12.14) for the FRAIL, SOF index, and TUG, respectively, with participant numbers of 11,838, 2,623, and 571, and 19, 3, and 4 included studies for these screening tools. Meanwhile, the pooled DORs were 35.03 (95% CI: 8.59, 142.90) and 13.66 (95% CI: 7.36, 25.36) for the CFS and KCL, with participant numbers of 459 and 556, and 3 included studies for each of these assessment tools.

For distinguishing the difference between primary care clinics and hospital-based outpatient clinics, there was no statistically significant difference in either the screening or assessment tools. In the screening analysis, the effect of setting was not significant (*p* =  0.58), with an estimated coefficient of 0.25 (95% CI: -0.64−1.15), and high residual heterogeneity (*I*^2^ = 90.19%), indicating substantial variability across studies. Furthermore, the moderator of setting accounted for no additional heterogeneity (R^2^ = 0%). In contrast, the assessment analysis showed a borderline significant effect for setting (*p* =  0.06), with an estimated coefficient of -1.62 (95% CI: -3.31−0.07), and moderate residual heterogeneity (*I*² = 31.23%). The inclusion of setting as a moderator explained a significant portion of the heterogeneity (R^2^ = 65.76%), suggesting that the variability in assessment tool performance may be partially explained by differences in settings.

### 3.6 Diagnostic accuracy ranking

Regarding the different cutoff points in the scales, Table 3 presents the diagnostic accuracy ranking of the five tools, with REFS excluded due to insufficient data for further analysis. For screening tools, the findings highlight that the FRAIL scale, using a cutoff point of 2, exhibited the highest Youden’s index at 0.60, with a sensitivity and specificity of 0.85 and 0.75, respectively. These results underscore the FRAIL scale, with a cutoff point of 2, as the optimal tool for frailty screening in older adults. For assessment tools, the findings indicate that the CFS, using a cutoff point of 5, exhibited the highest Youden’s index at 0.67, with a sensitivity and specificity of 0.75 and 0.92, respectively. These results suggest that the CFS scale, with a cutoff point of 5, is the optimal tool for frailty assessment in older adults.

**Table 3:** Ranking of frailty measurement scales based on Youden's index.

Index	Cutoff	Sensitivity	Specificity	Youden's index	Ranking
Screening					
FRAIL	1	0.83	0.66	0.49	3
FRAIL	2	0.85	0.75	0.60	1
FRAIL	3	0.65	0.90	0.55	2
TUG	7.7	0.68	0.61	0.29	7
TUG	8.2	0.76	0.63	0.39	5
TUG	10	0.78	0.67	0.45	4
SOF index	ND	0.18	0.99	0.17	9
SOF index	1	0.34	0.97	0.31	6
SOF index	2	0.32	0.97	0.29	7
Assessment					
CFS	4	0.83	0.81	0.64	2
CFS	5	0.75	0.92	0.67	1
KCL	3	0.7	0.78	0.48	4
KCL	7	0.8	0.78	0.58	3

### 3.7 Publication bias

Deek’s funnel plot asymmetry test indicated no significant publication bias in the studies for either the screening tools or the assessment tools, with p-values of 0.77 and 0.17, respectively, as shown in Appendix (Fig. 4A and B)

## 4 Discussion

Ongoing research in gerontology has emphasized the utility of validated instruments for the early identification of frailty [1,14]. The aim of our study was to evaluate the efficacy of various measurement tools in identifying frailty. Given the variations in physical performance, a key factor in detecting frailty, across different races and health conditions [18,19], this meta-analysis focused on the Asia-Pacific region, specifically within primary clinical practice and community-based settings. The results indicated that both screening and assessment tools exhibit comparable diagnostic performance in terms of sensitivity, specificity, and overall diagnostic accuracy. The two categories of scales reviewed demonstrated good, though not perfect, accuracy. Furthermore, the overall ability of each tool to accurately identify frailty in older adults ranged from moderate to high, suggesting that these instruments are reliable for both screening and assessing frailty in the Asia-Pacific population, particularly within primary clinical practice and community service settings.

Among the three scales used for screening, the results indicated that the FRAIL scale is the best-performing tool for distinguishing between frail and non-frail individuals, particularly in a study with a large number of participants. The confidence intervals for the FRAIL scale (9.66, 25.58) and the SOF index (3.80, 17.24) show limited overlap, suggesting a statistically significant difference in their performance. Additionally, the TUG scale (3.56, 12.14), had the lowest DOR, and its confidence interval does not overlap substantially with that of the FRAIL scale. Pairwise comparisons revealed that the FRAIL scale performed significantly better than both the SOF index (*p* =  0.03) and the TUG scale (*p* =  0.01) in distinguishing frail from non-frail individuals. Overall, the FRAIL scale demonstrated a significantly higher ability to identify frail individuals, as evidenced by its higher pooled DOR and statistically significant differences when compared to the SOF index and TUG scale (p < 0.05). These findings suggest that the FRAIL scale may be more suitable for frailty screening in larger populations. Our results align with the findings of Aprahamian et al. [51] and Woo et al. [52].

On the other hand, the results showed that the CFS outperformed the KCL as an assessment tool. The confidence interval for the CFS (8.59, 142.90) is considerably wider than that of the KCL (7.36, 25.36), reflecting greater uncertainty in the pooled estimate for the CFS due to the smaller number of participants and included studies [53]. This wide confidence interval suggests that the DOR for the CFS may be overestimated and should be interpreted with caution. The small number of studies (n = 3) and participants (n = 459 for the CFS and n = 556 for the KCL), however, limits the robustness of these findings. Small sample sizes and a limited number of included studies can lead to wide confidence intervals and an overestimation of the diagnostic accuracy of the tools, particularly for the CFS. Further research with larger sample sizes and additional studies is needed to validate these results. In summary, while the CFS demonstrated a higher pooled DOR (35.03, 95% CI: 8.59, 142.90) compared to the KCL (13.66, 95% CI: 7.36, 25.36), the wide confidence intervals and the small number of studies highlight the need for caution in interpreting these results.

While the spectrum of frailty severity and prevalence likely varies between non-healthcare community settings and clinic settings, our additional analyses, conducted with respect to the setting, revealed no statistically significant differences. However, we were unable to conclude the absence of statistically significant differences in frailty detection or prevalence between primary care clinics and hospital-based outpatient clinics due to the high residual heterogeneity among the studies of screening tools and the limited number of studies, with only one conducted in a community setting, among the studies of assessment tools. This contrast highlights that while the setting does not appear to influence the performance of screening tools (R² = 0%), it may play a more meaningful role in explaining the variability in assessment tool performance (R² = 65.76%). The high residual heterogeneity in the screening analysis (*I*² = 90.19%) suggests substantial variability across studies, potentially stemming from differences in study populations, methodologies, or frailty definitions. The borderline significance of the setting effect in the assessment analysis (*p* =  0.06) further indicates a potential trend that warrants additional investigation. Future studies should aim to include more balanced representations of community settings and adopt standardized frailty definitions to better elucidate the influence of setting on frailty detection and assessment.

Interestingly, the FRAIL scale, with a cutoff of 2, was identified as the optimal screening tool for detecting frailty in older adults, as our study demonstrated a sensitivity of 0.85 and a specificity of 0.75. While these values represent good diagnostic performance, they reflect comparatively lower sensitivity when measured against the Frailty Phenotype (FP), which the majority of studies use as the reference standard and which typically exhibits higher sensitivity for frailty identification. The FP, with its ≥3 cutoff, is designed to comprehensively capture frailty through a multidimensional assessment of physical and functional domains. In contrast, the FRAIL scale, as a simpler tool, prioritizes feasibility and practicality, particularly in community settings. This trade-off highlights the need for a lower cutoff (≥2) for the FRAIL scale to enhance its sensitivity, enabling it to better identify frailty while maintaining reasonable specificity. This adjustment ensures the FRAIL scale remains a practical and effective screening tool, especially in populations where a rapid or straightforward frailty assessment is required.

Moreover, a score of 2 on the FRAIL scale indicates pre-frailty, while a score of 3 corresponds to the original cutoff for frailty [2,54]. In our study, a score of 3 on the FRAIL scale exhibited lower sensitivity (0.65) but higher specificity (0.90) compared to a score of 2. A screening tool with low sensitivity leads to more false negatives, while one with high specificity reduces the occurrence of false positives [55]. Therefore, while frail cases identified using the FRAIL scale with a cutoff of 3 can be confidently classified as frail, frailty should not be ruled out in cases identified as non-frail. Thus, using the FRAIL scale with a cutoff of 2 would improve the overall accuracy of frailty detection in older adults.

Furthermore, in light of the recommendation in the Asia-Pacific CPGs for the management of frailty, published in 2016, that frailty diagnosis should be tailored to this population [15], it is noteworthy that although the majority of the studies included in this review were published between 2020 and 2022, the cut-off points for each frailty tool remained consistent with the original recommendations [[37], [38], [39], [40], [41], [42], [43], [44], [45], [46], [47], [48], [49], [50]]. For instance, four out of ten studies evaluated the FRAIL scale with a cutoff of 2 [39,41,43,50], while only three studies utilized a cutoff of 1 [38,45,46]. Nonetheless, the available evidence is insufficient to comprehensively assess the recommendation outlined in the Asia-Pacific CPGS. From this perspective, the present study provides substantial evidence to support the guidelines; however, further research is warranted to validate these findings.

Given the wide variations in frailty prevalence across the Asia-Pacific region [6,7], this study provides an up-to-date and accurate estimate of frailty prevalence among the community-dwelling population. Our study demonstrated that the pooled estimated prevalence of frailty and pre-frailty are 19.7% and 31.7%, respectively. Considering that high disease prevalence is typically associated with higher sensitivity and lower specificity in tests under evaluation [21], an adjusted cutoff may be more effective for identifying frailty, as differences in frailty prevalence across various cutoffs can affect the diagnostic accuracy of frailty [19,56]. Additionally, prevalence is a critical factor for calculating sample sizes in future interventions and informing decision-making processes for stakeholders and policymakers in healthcare agencies [11,14].

As with most studies, there are several limitations to acknowledge. Firstly, our study adhered to the 2017 Asia-Pacific CPGs framework for classifying tools as 'screening' or 'assessment.' While newer tools, such as the Frailty Phenotype Questionnaire and the Clinical Frailty Scale (CFS), have been introduced [[72], [73], [74]], evidence supporting their use as screening and assessment tools, respectively, remains limited. The CFS, although utilized in certain COVID-19 settings, is primarily designed as an assessment tool, while the Frailty Phenotype Questionnaire lacks sufficient validation for community-based use in the Asia-Pacific context. Moreover, we included only literature published in English, which may have introduced selection bias into the results. During the quality appraisal, we utilized the QUADAS-2 review framework and observed that most studies had unclear reporting in the domains of 'index test' and 'reference standard.' Although the applicability concerns in these areas were considered 'low,' these findings underscore limitations in the reporting of diagnostic studies, likely stemming from the fact that QUADAS-2 is primarily tailored for cross-sectional diagnostic studies. Furthermore, heterogeneity is a common issue in DTA reviews, particularly regarding diagnostic methods, thresholds, and reference standards. Some of the observed between-study heterogeneity may also be attributed to the inclusion of cross-sectional studies and small sample sizes, especially for tools used in assessment. Finally, the limited number of studies available for each tool within the Asia-Pacific population—except for the FRAIL scale—resulted in insufficient data for further analysis. Future studies should adopt standardized methods for frailty identification, ensure adequate sample sizes, and address these gaps more comprehensively.

## 5 Conclusions

The findings from this meta-analysis highlight the strengths and limitations of various frailty screening and assessment tools, particularly within the Asia-Pacific region. The FRAIL scale emerged as the best-performing screening tool for distinguishing between frail and non-frail individuals, especially in larger populations. Moreover, the FRAIL scale, with a cutoff of 2, shows high potential for early frailty detection. While the original cutoff of 3 enhances specificity, it compromises sensitivity, suggesting that a lower cutoff (e.g., 2) would improve overall diagnostic accuracy. Furthermore, this research provides a current and accurate estimate of frailty prevalence in the Asia-Pacific region, where the pooled estimated prevalence of frailty and pre-frailty were 19.7% and 31.7%, respectively. The high prevalence of frailty suggests that adjustments to the cutoffs of these tools may be necessary to improve their diagnostic utility. Overall, the results reaffirm the importance of validated frailty tools in primary clinical practice and community-based settings, while also emphasizing the need for larger, more comprehensive studies to validate these findings. The variability in frailty prevalence across the region and its implications for policy and clinical practice highlight the critical role of ongoing research in improving frailty identification and management.

## CRediT authorship contribution statement

Yi-Chen Wu: Conceptualization, Data curation, Formal analysis, Methodology, Project administration, Validation, Visualization, Writing – original draft. Chia-Te Chen: Conceptualization, Data curation, Investigation, Project administration, Validation. Shu-fen Shen: Data curation, Investigation, Validation, Visualization. Liang-Kung Chen, Li-Ning Peng & Heng-Hsin Tung: Conceptualization, Methodology, Validation, Writing – review & editing.

## Ethical approval

Not required.

## Funding

This research did not receive any specific grant from funding agencies in the public, commercial, or not-for-profit sectors.

## Data availability

The data that support the findings of this study are available from the corresponding author upon reasonable request.

## Declaration of competing interest

The authors declare that there have no known competing financial interests or personal relationships that could have appeared to influence the work reported in this paper.All authors discussed the results and contributed to the final manuscript and there is no conflicts of interest.

## Figures and Tables

**Figure:**
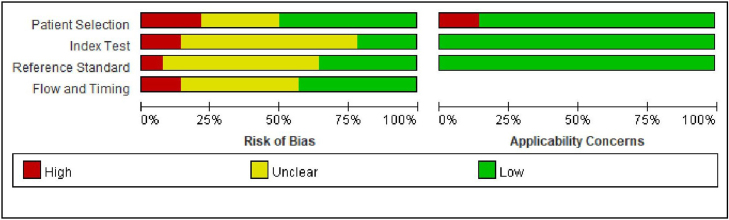


**Figure:**
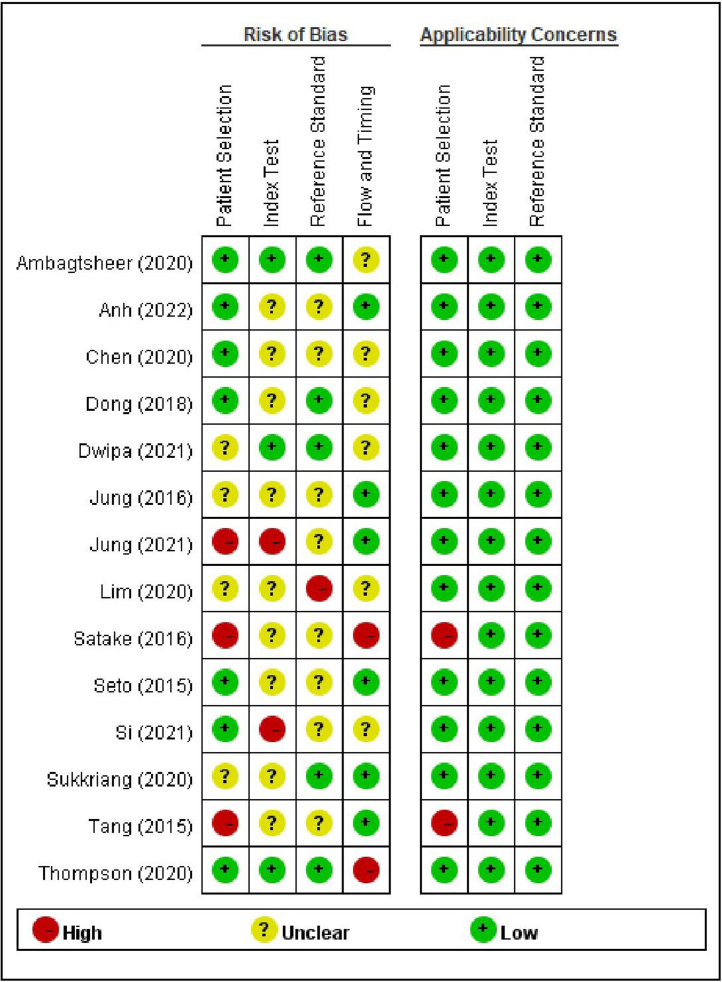


**Figure:**
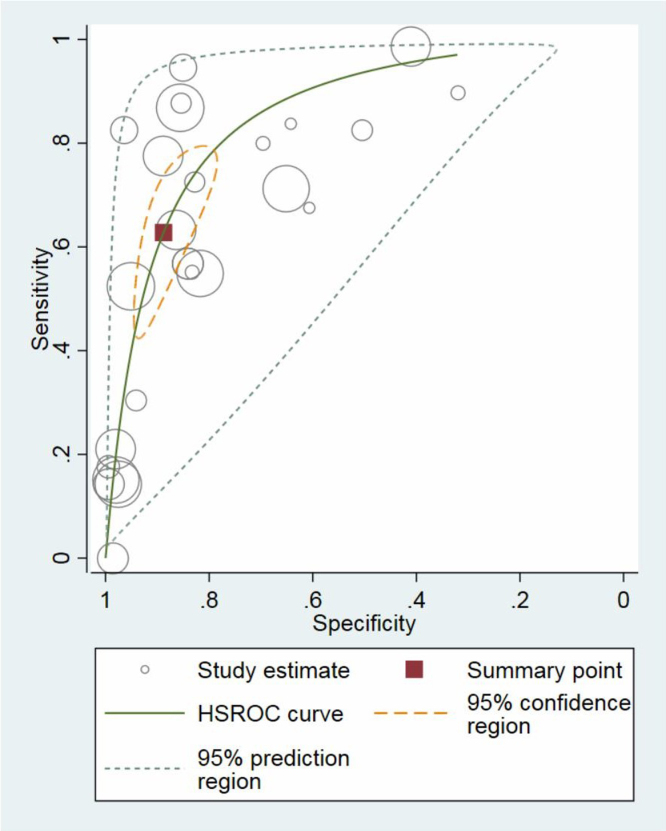


**Figure:**
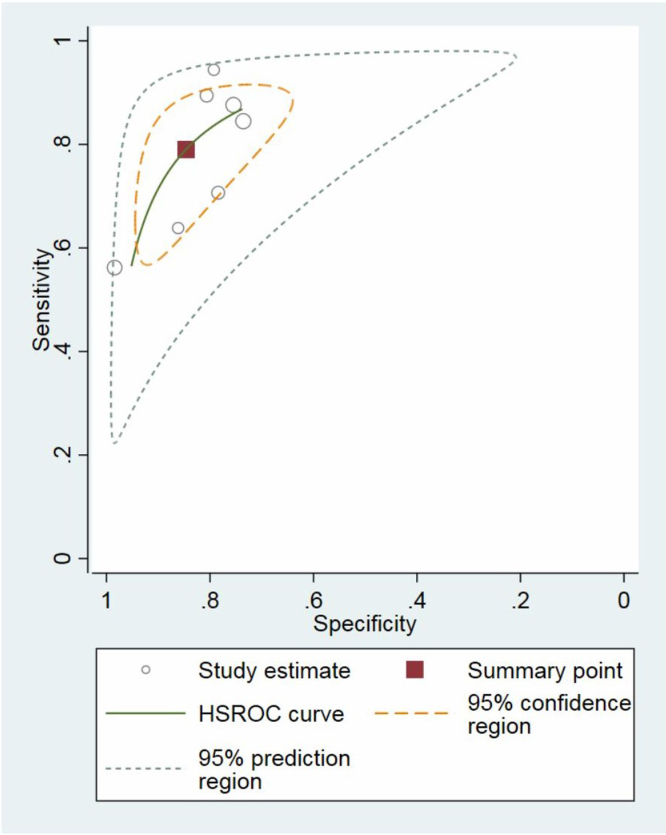


**Figure:**
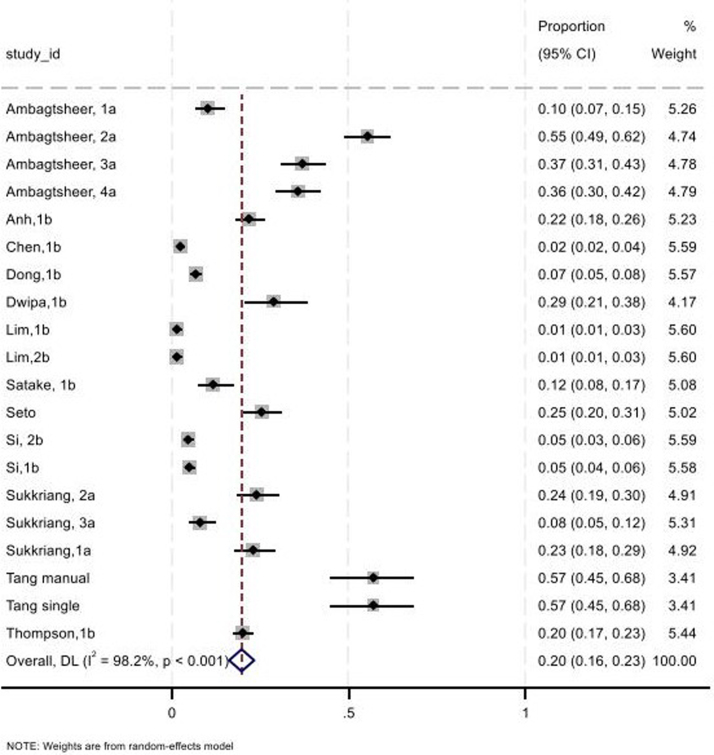


**Figure:**
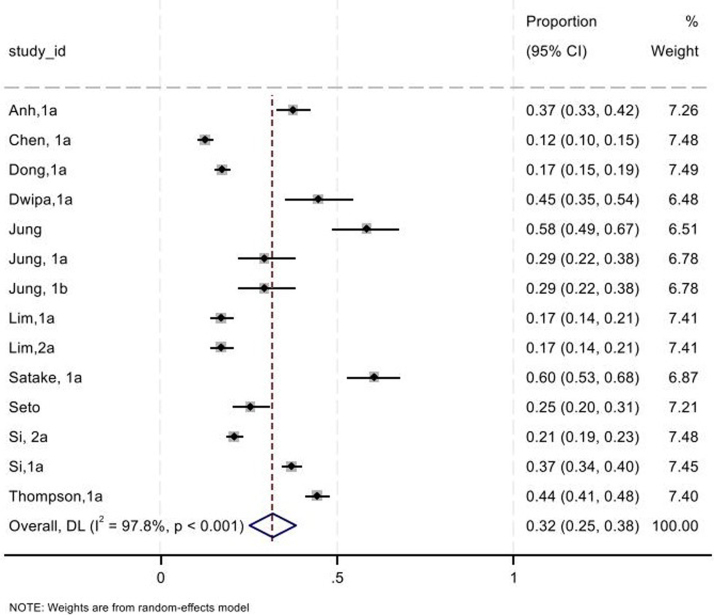


**Figure:**
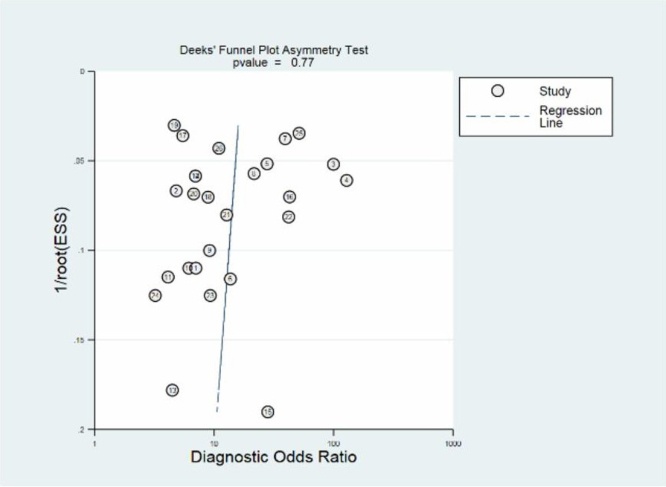


**Figure:**